# Epigenetic regulation in mammalian preimplantation embryo development

**DOI:** 10.1186/1477-7827-7-59

**Published:** 2009-06-05

**Authors:** Lingjun Shi, Ji Wu

**Affiliations:** 1 School of Life Sciences and Biotechnology, Shanghai Jiao Tong University, Shanghai, 200240, PR China

## Abstract

Preimplantation embryo development involves four stages: fertilization, cell cleavage, morula and blastocyst formation. During these stages, maternal and zygotic epigenetic factors play crucial roles. The gene expression profile is changed dramatically, chromatin is modified and core histone elements undergo significant changes. Each preimplantation embryo stage has its own characteristic epigenetic profile, consistent with the acquisition of the capacity to support development. Moreover, histone modifications such as methylation and acetylation as well as other epigenetic events can act as regulatory switches of gene transcription. Because the epigenetic profile is largely related to differentiation, epigenetic dysfunction can give rise to developmental abnormalities. Thus, epigenetic profiling of the embryo is of pivotal importance clinically. Given the importance of these aspects, this review will mainly focus on the epigenetic profile during preimplantation embryo development, as well as interactions between epigenetic and genetic regulation in these early developmental stages.

## Background

Starting from fertilization and ending with implantation, preimplantation embryo development can be divided into several well-orchestrated stages: fertilization, cell cleavage, morula and blastocyst formation. Understanding the stages of preimplantation development and the underlying regulatory molecular mechanisms is of pivotal importance for basic reproductive biology and for practical applications including regenerative medicine. To decipher the regulatory mechanisms, determining the global gene, RNA and protein expression patterns of early embryo is indispensable. Early attempts to do this provided data on protein expression patterns using comparative electrophoretic analysis with radiolabeled tyrosine and lysine [1] and RNA expression patterns were estimated using cDNA library analysis [2]. A series of subsequent studies reported modified or novel methods including polymerase chain reaction (PCR)-based differential display [3] and subtractive cDNA library construction techniques [4]. With the development of microarray technology, microarray analysis soon became one of the most powerful approaches, providing more comprehensive and precise global expression pattern data, especially on gene expression profiles [5-7]. Based on transitions in gene expression patterns in mouse embryos, preimplantation development can also be divided into several phases: phase I from fertilization to the 2-cell stage; phase II from the 4-cell to the 8-cell stage and phase III from the 8-cell embryo to the blastocyst stage. Phases I and II can also be called zygotic genome activation (ZGA) and mid-preimplantation gene activation, respectively [8]. At ZGA, some proteins originally formed in the stage of oogenesis remain after fertilization and contribute to the regulation of the next developmental processes.

Preimplantation embryo development is regulated both genetically and epigenetically. Based on the knowledge of genetic profiles, studies proceeded to elucidate epigenetic profiles in embryogenesis and their particular contributions to genetic changes. Epigenetics refers to a collection of mechanisms and phenomena that can cause a change in the phenotype of a cell or an organism without altering its DNA sequence [9]. DNA methylation, histone modifications, chromatin remodeling and various types of interfering RNA (RNAi) are all involved. These epigenetic events constitute a particular signature for each cell. During development, the epigenetic signature changes as the cell enters a special activity (e.g. differentiation or fertilization). Early studies focused largely on the effects of DNA methylation [10,11] and the regulation of histone modifications [12,13] on preimplantation embryo development. Further studies have added chromatin organization (core histone variants) [14] to this epigenetic regulation network, making it more complex, intriguing and – more importantly – making the start of life more mysterious and beautiful.

## Epigenetic regulation

The mature spermatozoon and oocyte acquire specific and different epigenetic marks during gametogenesis. Besides imprints, in the spermatozoon somatic linker histones are replaced by testis-specific variants and then most histones are replaced with protamines [15] whereas in the oocyte the level of H3 lysine(K)4me3 may be higher because of the enter of meiosis [16]. Parental imprints derived from gametogenesis are retained faithfully in the zygote as well as some other marks. However, others are reprogrammed dynamically to meet the need of becoming totipotent. As development proceeds, epigenetic marks undergo reprogramming again to adopt embryo to be fit for further differentiation. Epigenetic reprogramming includes DNA methylation, histone modifications and the formation of histone variants.

### DNA methylation

DNA methylation is a covalent chemical modification of DNA, which transfers the methyl group of the coenzyme S-adenosyl-L-methionine to cytosine residues of CpG dinucleotides and is one of the main epigenetic events [17]. To estimate the DNA methylation state, many detection methods have been developed. Methylated sequences can be differentiated by sodium bisulfate DNA modification or methylation-sensitive restriction enzyme digestion, followed by PCR or hybridization methods (Southern blotting and microarrays). Recently, there has been more focus on genome-wide DNA methylation profiling, which is determined by microarrays, high-performance liquid chromatography (HPLC) and restriction landmark genomic scanning (RLGS) [18]. Based on these technologies, the first high-resolution DNA methylation profile of Arabidopsis thaliana genome has now been determined [19], opening the way for 'methylomics'.

DNA methylation is an important epigenetic event, regulating chromatin structure and gene expression in many developmental processes including gene imprinting, X chromosome inactivation and embryogenesis [20]. To maintain or establish methylated DNA, DNA methyltransferases (Dnmts) are indispensable [21]. These can be divided into three classes: Dnmt1, Dnmt2 and Dnmt3 (containing three subclasses Dnmt3a, Dnmt3b and Dnmt3l). Dnmt1 is the main methyltransferase by far and its exceptional preference for hemimethylated DNA indicates its role in the maintenance of methylated status during DNA replication. Dnmt2 is a methyltransferase but its function is obscure and controversial. Dnmt3a and Dnmt3b are both involved in de novo methylation processes but with different preferences. Thus, Dnmt3a prefers to act on unmethylated DNA while Dnmt3b can assume its role on both hemimethylated and unmethylated DNA. Dnmt3l is devoid of enzymatic activity, although its role as a regulator of proper methylation is still pivotal [21,22]

**Figure 1:**
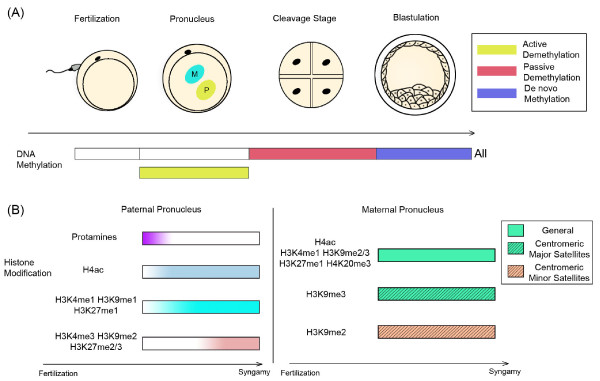
**General view of the main epigenetic reprogramming pathways in preimplantation development**. (A) DNA methylation reprogramming in the mouse embryo. Active demethylation happens in paternal PN(pronucleus) followed by passive demethylation of the entire genome during cleavage stage. Then comes de novo methylation when the embryo commences cell differentiating. (B) Histone modification reprogramming, from fertilization to syngamy. In the paternal PN, sperm nuclear protamines are gradually replaced by highly acetylated histones, which are immediately substituted by monomethylated ones. Dimethylated and trimethylated histones appear later. In the maternal PN, all the modifications listed can be detected in this period. H3K9me3 is found in centromeric major satellites, whereas H3K9me2 is found in minor satellites.

During preimplantation stages in embryogenesis, DNA methylation experiences dynamic changes. In the mouse embryo, the paternal and maternal pronuclei (PN) are formed following fertilization. There is active demethylation in the paternal PN [23], whereas DNA is passively demethylated during the later cleavage stages for the entire genome [24]. The embryo then undergoes the first cell differentiation process, accompanied by de novo DNA methylation, which gives rise to stable silencing of genes related to the maintenance of pluripotency [25]. The level of methylation in the inner cell mass (ICM) of the blastocyst is higher than that in the outside cells, corresponding to different cell fates: the ICM will differentiate into somatic cell lineages whereas the outer cells will form the placenta (see reviews [26,27]) (Fig. 1A). However, these methylation changes do not always tell the same story. Even in laboratory mice, variations may arise [28], probably caused by inherent genotypic variability. Moreover, this general model is also challenged by controversial data obtained from other mammals including the sheep [29], rabbit [30] and human [31]. This suggests two possibilities: the first is that there is no functional relevance between methylation changes and development; the second is that such methylation changes are species-specific and no general rule can be drawn. However, we should still endeavor to confirm either of them, or put forward alternative models.

Despite the dramatic changes in global methylation levels, the methylation status of imprinted genes must remain. A recent study showed that Dnmt3a and Dnmt3b are dispensable for the maintenance of DNA methylation status during embryonic cleavage stages and Dnmt1 alone is sufficient to accomplish the job at most of the imprinted loci except for the Rasgrf1 differentially methylated region (DMR) [32]. Dnmt1s, an isoform of Dnmt1, was found to be present in association with chromatin in the nucleus throughout preimplantation development and was related to the maintenance of methylation in particular genomic regions such as repetitive intracisternal A-type particle (IAP) sequences and the H19 imprinted locus [33]. Zfp57, a maternal zygotic effect gene, has also been found to be related to the maintenance of both maternal and paternal imprints [34].

Methylation alterations also flourish at levels besides global changes. Dnmt1o, another isoform of Dnmt1, is expressed predominantly during oogenesis and early preimplantation development. Dnmt1o has a tissue-dependent DMR (T-DMR) and its DNA methylation status changes stage-by-stage. Thus Dnmt1o, as a participant in the epigenetic processes, is also influenced by methylation in terms of its expression level [35]. Moreover, analysis of mRNA expression patterns of methyltransferases, methylcytosine-binding proteins for chromatin modification and base excision repair enzymes (which might in turn be involved in active demethylation) [36] showed that even within one embryo, different cells can show divergent expression patterns. In other words, their epigenetic profiles differ from each other. This complicates the analysis further, as now even single cells need to be given their own 'identity card'. Global analysis can show the situation in an average level, but it overlooks the regional specificity. DNA methylation profiling of a single cell might show additional information and give indications of its fate in differentiation.

DNA methylation is an important and irreplaceable event in preimplantation development. It undergoes dynamic changes not only globally but also in region-specific or locus-specific manners. Furthermore, different cells in a same embryo were found to possess different expression patterns of methylation-related genes, indicating the importance of the construction of single-cell-specific profiling besides that of entire embryos. Analyzing the methylome may help to provide more data about temporal and/or spatial DNA methylation status and complete the story of DNA methylation reprogramming.

### Histone modifications and chromatin remodeling

Histone modification is another covalent modification that is involved in the regulation of gene expression. Histone modifications include acetylation, methylation, phosphorylation, ubiquitylation and sumoylation [37]. These can be divided into two groups: small chemical groups involving the first three processes, and much larger peptide groups involving the latter two [38]. Besides acetylation and methylation, other modifications may also have their peculiar roles in preimplantation development [39,40]. However, acetylation and methylation may be more ubiquitous, so more focus is put on these processes and changes in their status during development have been delineated clearly [26]. Additionally, histone modification also interacts with DNA methylation to produce further epigenetic regulation. Suv39h1 histone methyltransferase (HMTase) directed H3K9 trimethylation was shown to be required for recruiting Dnmt3b-dependent DNA methylation to pericentromeric repeats [41]. Also, DNA methylation recruits the methyl-binding domain containing proteins (MBDs) and assembles histone deacetylase multiprotein repressory complexes (MeCP1 and Mi2/NuRD) [42]. However, the dynamics of such histone modifications might be more complex and flexible, for the reason that DNA methylation brings about more stable silencing of gene expression than histone modifications do.

Generally, histone modifications also undergo dynamic changes during preimplantation development. From fertilization to syngamy, acetylated lysine (H4ac), the methylated histones H3K4me and H3K9me2/3, H3K27me1, H4K20me3 are discernable in the female PN [43-45]. These are associated with active chromatin states and with repressive chromatin organization. Moreover, H3K9me3 is found in centromeric major satellites, whereas H3K9me2 is found in minor satellites [44]. During formation of the male PN, the protamines begin to be exchanged for highly acetylated histones [43]. However, immediately afterwards the histone acetyl groups are substituted by monomethyl groups and H3K4me1, H3K9me1 and H3K27me1 are detectable [46]. Moreover, in contrast with the monomethylated histones, dimethylated and trimethylated histones appear later [44] (Fig. 1B) (see review [26]). Meanwhile, the level of histone arginine methylation (H4R3me and H3R17me), an activating mark of gene expression, also decreases as lysine acetyl modification does [47].

Specifically, histone modifications change in stage- and cell type-specific manners and assume their roles as switches of gene expression, by which gene expression can be precisely controlled according to necessity. For example, during differentiation Oct4 and nanog are silenced progressively with the first step being H3K9 methylated [48]. In the blastocyst, the ICM and trophectoderm have different histone modification profiles [49]. H3K9 acetylation and heterochromatic histone methylation are reestablished preferentially in the ICM. H3K27me1/2/3 can be detected in both cell lines but are more predominant in the ICM, and H3K27me2/3 only appears in the inactive X chromosome in the trophectoderm [49]. H3K9me2/3 show even activity levels in the ICM and trophectoderm and H3K9me3 marks heterochromatic foci [49] (see review [26]). These differences result from the different fate of ICM and trophectoderm. ICM and trophectoderm have different gene expression profiles. Therefore, their epigenetic profiles also differ accordingly for precise control.

Genes related to histone modifications include HMTases (e.g. G9a, ESET and Suv39h) [37], histone deacetylases (e.g. HDAC1, HDAC2 and HDAC3) [50], etc. The enzymes ESET and Suv39h both catalyze H3K9 methylation and build a binding site for HP1 (Heterochromatin protein 1) to form heterochromatin, however Suv39h specifies parental asymmetry in constitutive heterochromatin [51] and ESET goes further to be needed for maintaining cell viability [52]. Additionally, although HDAC1 was found to be a major deacetylase in preimplantation development and mainly functions as a H4K5 deacetylase, HDAC1 knockdown does not affect the global transcription rate, suggesting its role for a subset of genes but not all [53]. Other HDACs might also play some roles, however there is little evidence yet. Histone acetylases and deacetylases are also involved in the modification of chromatin along with ATP-dependent chromatin remodeling protein complexes (SWI/SNF, ISWI and Mi-2/NuRD), which alter histone/DNA interactions via ATP hydrolysis to make DNA more accessible to various factors including transcription factors (TFs) [54]. SRG3, Brg1 and Ini1 are the three core subunits of the SWI/SNF complex. Maternal Brg1 was found to be a good candidate for being involved in ZGA [55] as was SRG3, whose expression pattern was found to parallel those of TFs such as Sp1 and TBP [56], reinforcing the important roles of chromatin remodeling protein complexes in preimplantation embryogenesis.

Histone modification reprogramming is more complex than DNA methylation and no general model can be drawn from the accessible experimental data so far. However, similar to DNA methylation, histone modification changes dynamically during preimplantation development in stage- and cell type-specific manners, which are required for the precise regulation of gene expression. As participants in histone modification, the histone acetylases and deacetylases are also involved in chromatin remodeling and help to pave the path for various factors to the DNA.

### Histone variants

Histones are building blocks of nucleosomes, each consisting of eight core histone proteins (two each of H3, H4, H2A and H2B) to form an octamer [57]. In addition, histone protein H1 functions as a linker [57]. A set of histone variants has been found in mammals including H3 variant CENP-A [58], H3.3 [59] and H2A variant H2AZ [60]. CENP-A involves in the formation of the foundation for kinetochore assembly [58]; H3.3 incorporation always happens at active genes [59]; H2AZ functions in transcriptional regulation and chromosomal segregation [60]. During preimplantation development, histone variants also play important roles. Linker histone H1 has six variants in which H1^o ^shows obvious difference from other five termed somatic H1 subtypes [61]. H1^o ^was reported to be expressed in the oocyte and the role of somatic H1 declines during early cleavage stages, which may be related to the establishment of regulated embryonic gene expression [61]. For macroH2A, the maternal stock is removed before syngamy and the embryo proceeds with cell division until the 8-cell stage after which macroH2A protein expression reappears and persists into the blastocyst stage. MacroH2A was found to be largely related with expression inhibition and the facultative heterochromatin of inactive X chromosomes [62] while in contrast with macroH2A, another histone variant, H2A^Bbd^, which has a role in facilitating transcription [63], is depleted [64]. The emerging view from these studies is that histone variants when deposited into the nucleosomes, provide a differentiation of chromatin and add to the epigenetic regulation of preimplantation development.

### Noncoding RNAs

Since the discovery of the first miRNA in 1993 [65,66], miRNAs have attracted tremendous interest. MiRNAs are short ncRNAs with 20–24 nucleotides (nt) that regulate gene expression either by cleaving target mRNAs or by blocking mRNA translation. Since lin-4 was first identified in regulation of the developmental timing of cell fate in larval stages of Caenorhabditis elegans [65,66], miRNAs have been shown to function in various stages of development including preimplantation development. During miRNA biogenesis, Dicer (ribonuclease type III) functions to cleave pre-miRNAs into mature miRNAs [67] and these are incorporated into a complex termed RNA-induced silencing complex (RISC) in which proteins with activities such as helicase, exonuclease and the protein Argonaut (Ago) facilitating RNA recognition include. RISC then goes on to degrade mRNAs or to block their translation [68]. Dicer deficiency is lethal during mouse embryogenesis, leading to a lack of detectable stem cells and an acute loss of proliferation potential [69]. Ago2 is required for development through the ZGA period. During ZGA, maternally-derived transcription products are destroyed. This suggests that RNAi-mediated machinery may be involved in the degradation of a proportion of maternally-derived transcription products [70]. Evidence also comes from recent microarray analysis [71], revealing the expression pattern of miRNAs and their stage-specific functions. In all, 97 miRNAs have been analyzed and can be divided into stage-specific and non stage-specific groups. During ZGA, miRNAs may mainly participate in the degradation of maternal messages [71]. During morula stage compaction, miRNAs may be mainly involved in cell adhesion [71]. MiRNAs may also play roles in differentiation during the blastocyst stage and a cluster of miRNAs from miR-290 to miR-295 were found to be embryonic stem (ES) cell-specific, which may be associated with the maintenance of pluripotency [71]. Thus, function-specific miRNAs are expressed to fulfill the needs of developmental processes and miRNAs, like other genes, are correctly regulated temporally and spatially by upstream regulators.

Besides short ncRNAs, long ncRNAs also conduct important development events in their own ways [72,73]. Long ncRNAs are involved in the development of the germline, eye and brain. Though not yet clearly elucidated, there is mounting evidence to show that they have major roles in regulating epigenetic trajectories in animal development. Recent results [74,75] confirm this idea further: GAL10-ncRNA was found to be a modulator of methylation and histone deacetylation in the yeast GAL gene cluster [74] and GHRLOS, a ncRNA candidate, might have regulatory and functional roles in the ghrelin hormone response [75]. During preimplantation development, long ncRNAs are involved in gene silencing [76] and gene imprinting [77]. The Air ncRNA is required for allele-specific silencing of Slc22a3, Slc22a2 and Igf2r genes in the mouse (Slc22a3, Slc22a2 are organic cation transporter involved in organ-specific transport of various molecules [78] and Igf2r functions in lysosome biogenesis and growth suppression [79]). It accumulates at the Slc22a3 promoter and recruits H3K9 methyltransferase G9a to fulfill imprinting [76]. Another mouse imprinted domain, Kcnq1, is located on the distal arm of chromosome 7 and contains several imprinted genes. Repression of these genes is regulated by a non-coding antisense transcript, Kcnq1ot1, which is paternally expressed, but maternally repressed by DNA methylation derived from the oocyte. The expression of Kcnq1ot1 then triggers repression [77]. This regulation pattern is largely similar to X chromosome inactivation, in which the ncRNA Xist coats the X chromosome and inactivates it [80]. When initiated, both will recruit other epigenetic elements and undergo further silencing, which will be described in detail below.

### X chromosome inactivation (XCI) and the Kcnq1 imprinted domain

**Figure 2:**
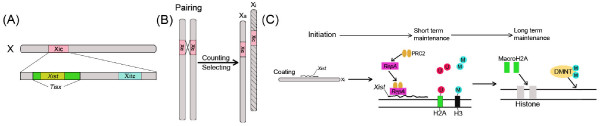
**X chromosome inactivation and regulatory mechanisms**. (A) The Xic region of the X chromosome and several regulatory elements in this region. Xist loci encodes a ncRNA which mediates the initiation of XCI. Xist is negatively regulated by another ncRNA, Tisx, which is complementary to Xist. Xite is a special upstream enhancer of Tisx. (B) By pairing at the Xic region, X chromosomes communicate and counting, selecting are then undergone to make sure that only one X chromosome remains active. Xa, active X chromosome; Xi, inactive X chromosome. (C) Mechanisms from initiation of XCI to the short- and long-term maintenance of silencing status. After selection, Xist coats the Xi chromosome to initiate XCI. A ncRNA (RepA) then usher polycomb repressive complex 2 (PRC2) to the Xi chromosome, recruiting histone modification changes such as H2A-K119 ubiquitination (U) and H3K27 methylation (M). Histone modifications bring about short-term silencing followed by histone variant incorporation (macroH2A) and de novo DNA methylation, mediating long-term silencing.

X chromosome activity changes dynamically during preimplantation development and its inactivation is a combination of epigenetic events including DNA methylation, histone modifications and RNA-mediated silencing (Fig. 2). In female embryos with two X chromosomes, the paternally-derived X chromosome is inactivated during cleavage stages and persists this status in the trophectoderm and subsequently in the placenta [81]. However, in the ICM this inactivation reverses and after differentiation one of the two X chromosomes is selected stochastically to be inactivated [82]. XCI is triggered by a ncRNA Xist which is transcribed from a key locus on X chromosome termed the X inactivation centre (Xic) [83]. Xist has been found to be negatively regulated by Tisx. Tisx, as its name indicates, is also a ncRNA that is complementary to Xist. Besides, Tisx is in turn regulated by Xite, a special upstream enhancer of Tisx [84]. Several studies have shown that Tisx transcription brings about changes to chromatin, which in turn regulate Xist transcription. Furthermore, Tisx-regulated Xist silencing seems to be initiated by changes in histone modifications and to be maintained by Tisx-dependent recruitment of Dnmt3a to the promoter of Xist for stable silencing [85-87]. During random selection for XCI, two X chromosomes crosstalk by pairing on their Xic regions. This is mediated by Tsix and Xite and a choice is then made via this process followed by initiation of XCI [88,89]. A recent study shed some light on the initial spread of XCI. A 1.6 kb ncRNA (RepA) was discovered within Xist. It serves to usher polycomb repressive complex 2 (PRC2), as the direct target of RepA, to the X chromosome [90]. Moreover, the maintenance of silencing might be mediated by PRCs (PRC1 and PRC2) [91], which can lead to histone modification changes such as H2A-K119 ubiquitination and H3K27 methylation. This suggests that RepA serves to link initiation and maintenance. In addition to the factors mentioned above involved in the early maintenance of XCI, long-term maintenance machinery should exist. This includes the participating of histone variants (incorporation of macroH2A) and de novo methylation of the promoters of X-linked genes [91]. XCI is complicated as the inactivation status changes dynamically and the epigenetic interactions involved in different phases of XCI – initiation, short and long-term maintenance of silencing status – also change dynamically. Although XCI has been elucidated in the whole, controversies still exist and many questions still need to be answered. For example, X chromosome crosstalk-pairing and random selection for active/inactive X chromosome (Xa/Xi) may be earlier than previously proposed according to the recent studies [92,93]. And, XCI may have another layer of regulation where both X-silencing on Xi and X-upregulation on Xa are involved [94,95].

As discussed above, the imprinting process of the mouse Kcnq1 imprinted domain is almost similar to XCI. Imprinting is initiated by a ncRNA Kcnq1ot1 from the 2-cell stage during preimplantation development and is maintained by altering the status of histone modifications including H3Ac, H3K4me2, H3K9me2 and H3K27me3, resulting in allele-specific (paternal) and region-specific (placenta/embryo) gene silencing [77].

In conclusion, single epigenetic events (DNA methylation, histone modification, RNAi, etc.) are able to play important roles as switches in the regulation of gene expression, but they always interact to accomplish their responsibilities. Combinations of several epigenetic events have evolved to conduct even more complex silencing such as X chromosome inactivation and gene imprinting, which makes it even more difficult to understand the epigenetic regulation network. The status of epigenetic modification should be examined, but this is not the end of the task. The interactions and the hierarchies between each event involved need to be determined carefully. What is more, the dynamics of epigenetic regulation is another area to be elucidated, as this is necessary for measuring gene expression profiles.

### Interactions between epigenetic and genetic regulation

**Figure 3:**
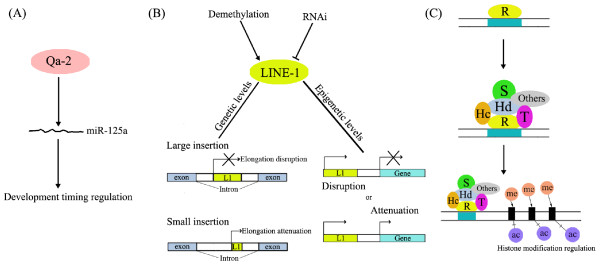
**Some examples of interactions between genetics and epigenetics**. (A) The Ped gene protein product Qa-2 affects the level of an epigenetic element (miR-125a) to participate in the interaction. (B) The LINE-1(L1) retrotransposon expression is precisely regulated by epigenetic mechanisms (negatively by RNAi and positively by demethylation), while its expression and activation can effect gene expression both genetically and epigenetically. When genetically, large and short insertion cause elongation disruption or attenuation. When epigenetically, expression of L1 disrupts or attenuates gene expression without insertion. (C) Ronin (R) binds DNA directly; once bound it recruits HCF-1 (Hc) and other proteins that are related with transcriptional repression or histone modifications, among which are THAP7 (T), sin3A (S) and HDAC3 (Hd), to form a large protein complex and regulate histone modifications (methylation and deacetylation) so as to affect gene expression.

**Figure 4:**
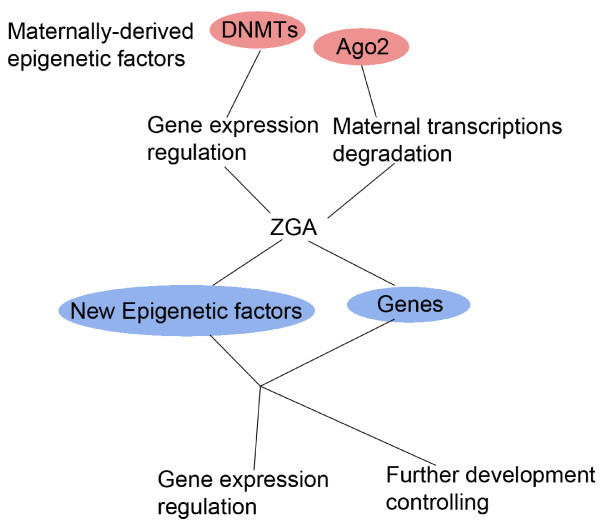
**Epigenetic and genetic interactions during early preimplantation development**. After fertilization, some maternally-derived epigenetic participants (e.g. DNMTs, Ago2) remain to be involved in gene expression transition(ZGA) and alter the gene expression status, leading to the expression of more epigenetic participants and other genes. These newly expressed ones are then added to further regulate the gene expression and developmental events.

Epigenetic regulation and genetic regulation are strictly interrelated. On one hand, every epigenetic event needs certain enzymes (e.g, Dnmts for DNA methylation, HMTases and HDACs for histone modifications) or protein complexes (e.g, PRCs, MeCP1). Therefore, the expression of the main epigenetic participants should be initiated and then epigenetic regulation could be on the way. The expression pattern of some epigenetic participants are consistent with globe gene expression transition (ZGA) while some are not. HDAC1 belongs to the former ones, which initiates expression at the single cell stage and its expression level increases gradually until the blastocyst [53]. Eset belongs to the latter ones. Its expression will not be initiated until the blastocyst stage when maternal ESET becomes exhausted [52]. On the other hand, epigenetic regulation always assumes its responsibility in cooperation with genetic regulation. During preimplantation development, the embryo will commence differentiating into two parts, ICM and trophectoderm. ICM remains pluripotent and trophectoderm loses it. Therefore, Oct4, as a key transcription factor of pluripotency, its expression needs to be precisely regulated. Sall4 binds with the enhancer of Oct4 and activates the expression of Oct4 [96], Tead4 controls the expression of the transcription factor Caudal-related homeobox 2 (Cdx2) and then impedes the expression of Oct4 [97]. Meanwhile, the status of methylation and histone acetylation in the Oct4 enhancer/promoter is altered to regulate its expression [98]. Oct4 also acts to promote the expression of another key transcription factor of pluripotency, Nanog, by binding to the proximal region of Nanog promoter [99]. Nanog expression is also regulated epigenetically. In the cells of ICM, histone H3 and H4 are highly acetylated and H3K4 is hypermethylated at the Nanog locus [100]. Finally, epigenetic regulation is always interlaced with genetic regulation in a hierarchy to form a regulatory network. For example, the mouse preimplantation embryo development (Ped) gene product, Qa-2 was recently found to affect the level of miR-125a [101] which is involved in the regulation of timing of early mouse embryo development (Fig. 3A). This gives powerful evidence of genetic and epigenetic interactions during preimplantation development in which LINE-1 retroposons [102-104] and Ronin [105] are also involved. LINE-1 retroposons expression is precisely controlled by epigenetic events while LINE-1 can alter the gene expression level both genetically and epigenetically [102-104] (Fig. 3B). Ronin associates with DNA and then recruits some proteins to alter the histone modification status [105] (Fig. 3C). Also, after fertilization, some maternally derived epigenetic participants remain and are involved in gene expression transition, which alter the gene expression status and combine with the newly expressed epigenetic participants and other genes to further regulate the gene expression and development (Fig. 4).

## Conclusion

Preimplantation embryo development is a complex process, which is regulated not only genetically but also epigenetically. With the endeavor of several generations of scientists, the code of the beginning of life has been deciphered gradually. Microarray analysis has broadened our knowledge of gene expression profiles in the preimplantation embryo. Certain gene expression profiles help to unravel the mystery of these developmental stages, which further promote research on the epigenome. We are still in the early exploratory stages of epigenomics. Studying the epigenome is intriguing and important because minor defects can lead to serious human diseases. Thus, several syndromes are associated with imprinting defects during preimplantation development [106-108]. Many aspects of epigenomics are now understood and many exciting studies have been done including research on the methylome. This is of significant clinical importance and can provide some biomarkers of certain human diseases, especially cancer [109]. Histone methylation is also important, recent study [110] indicated that histone methylation can be epigenetic mark for leukemia. Although a general model of DNA methylation status has been built using the mouse embryo model, is this a general paradigm or is the changing pattern of DNA methylation species-specific? Although the status of common histone modifications has been examined, what about the others: do they play key roles in these developmental stages? Although miRNAs and other longer ncRNAs are broadly related with developmental events, there is little evidence on the exact roles of certain ncRNA types. Moreover, more focus should be put on the delineation of epigenetic pathways and interaction, not just on single epigenetic event. We need to examine the interactions between different epigenetic events and the interactions between epigenetic regulation and genetic regulation. Global profiling sometimes cannot tell the whole truth, so single cell profiling is essential to construct a molecular regulation blueprint and help us in dealing with human diseases involving imprinted genes.

## Competing interests

The authors declare that they have no competing interests.

## Authors' contributions

LS drafted the manuscript. JW initiated and mentored the study in addition to providing a valuable framework for drafting the manuscript and revised it. All authors read and approved the final manuscript.
